# Prognostic value of CD133^+^ CD54^+^ CD44^+^ circulating tumor cells in colorectal cancer with liver metastasis

**DOI:** 10.1002/cam4.1241

**Published:** 2017-11-03

**Authors:** Chao Fang, Chuanwen Fan, Cun Wang, Qiaorong Huang, Wentong Meng, Yongyang Yu, Lie Yang, Jiankun Hu, Yuan Li, Xianming Mo, Zongguang Zhou

**Affiliations:** ^1^ Department of Gastrointestinal Surgery West China Hospital Sichuan University Chengdu China; ^2^ Institute of Digestive Surgery State Key Laboratory of Biotherapy West China Hospital Sichuan University Chengdu China; ^3^ Laboratory of Stem Cell Biology State Key Laboratory of Biotherapy West China Hospital Sichuan University Chengdu China

**Keywords:** CD133^+^CD54^+^CD44^+^ cellular subpopulation, circulating tumor cells, colorectal cancer, liver metastasis, prognostic value

## Abstract

In the previous study, we had showed the expression of CD133^+^ CD54^+^ CD44^+^ cellular subpopulation of circulating tumor cells (CTCs) was significantly associated with liver metastasis of colorectal cancer (CRC). This study aimed to explore whether this subpopulation of CTCs have a prognostic value in CRC patients. Flow cytometry was used to detect the expression of cellular subpopulations of CTCs with CD133, CD54, and CD44 in 152 CRC patients, between December 2013 and October 2014. The impact of clinicopathological factors and the expression of cellular subpopulations of CTCs on overall survival were then analyzed. CRC patients with liver metastases who underwent resection of the primary tumor accompanied by surgical treatment for metastasis had a better survival than other patients (*P *<* *0.001). The liver metastatic CRC patients with high expression of CD133^+^ CD54^+^ (*P *<* *0.001), CD133^−^ CD54^+^ (*P* = 0.004), and CD133^+^ CD44^+^ CD54^+^ (*P *=* *0.003) cellular subpopulations of CTCs had a worse survival than those patients with low expression. Multivariable survival analyses identified carcinoembryonic antigen levels (hazard ratio [HR] = 3.056; 95% confidence interval [CI] = 1.354–6.897; *P *=* *0.007), treatment strategy (HR = 0.212; 95% CI = 0.056–0.808; *P *=* *0.023), and CD133^+^ CD44^+^ CD54^+^ cellular subpopulation of CTCs (HR = 6.459; 95% CI = 1.461–28.558; *P *=* *0.014) as independent prognostic factors for CRC patients with liver metastasis. CD133^+^ CD44^+^ CD54^+^ cellular subpopulation of CTCs has a prognostic value in CRC patients with liver metastasis, especially in the survival of CRC patients with liver metastasis who did not undergo surgical treatment for metastasis.

## Introduction

Owing to the identification of risk factors, introduction and dissemination of screening tests and improvements in treatment, mortality rate of colorectal cancer (CRC) have been declining in recent years; however, the existence of liver metastasis is still one of the most important prognostic factors for CRC patients 1, 2, 3. As reported in various studies, approximately 50–60% of CRC patients will develop a distant organ metastasis during the progression of the disease and it is still one of the major causes of death in CRC patients 4, 5. Increasing evidence indicates that synchronous metastatic colorectal liver disease is associated with a more disseminated disease state and worse prognosis than metachronous metastatic colorectal liver disease 6, 7, 8. Thus, the evaluation and identification of new prognostic factors for synchronous liver metastasis provide the chance to explore new effective treatment strategies and to improve the survival of CRC patients with liver metastasis 6, 7, 9.

Circulating tumor cells (CTCs) are defined as tumor cells circulating in the blood and metastasis‐initiating cells (MICs) are referred as a fraction of CTCs having the capability to metastasize 10, 11, 12. MICs are critical to understand the biological mechanism of metastasis and are an important factor in identifying new treatments to increase the survival of metastatic CRC patients 11, 12, 13. The increasingly advanced and sensitive technologies together with the increasing cell surface markers have provided the opportunity to study the CTCs or MICs in detail 14, 15, 16, 17. In our previous study, we showed that the rare CD54^+^CD44^+^ cellular subpopulation in the tissues of rectal cancer patients possessed the cancer initiating potency because this cellular subpopulation exhibited a self‐renewal capability, potential epithelial–mesenchymal transition characteristics, and possessed strong tumorigenic capability in vivo 18. We also had found that CD133^+^ cellular subpopulation could be used as a baseline to select and isolate CTCs in the peripheral blood of CRC patients using fluorescence‐activated cell sorting (FACS). We then reported that the expression of CD133^+^CD54^+^CD44^+^ cellular subpopulation of CTCs was significantly associated with liver metastasis in CRC patients 19. In this study, we aimed to explore whether this cellular subpopulation in the peripheral blood has a prognostic value for CRC patients, especially those with liver metastasis.

## Materials and Methods

### Sample collection, preparation, and detection of CTCs

Peripheral blood samples were obtained from CRC patients attending our department and an informed consent was obtained from all the individuals. Peripheral blood samples were collected and prepared as per the protocol described in our previous report 19. In detail, CTCs from cell suspensions were characterized by multiparameter flow cytometry. The antibodies used in this study included anti‐human CD133‐APC, CD44‐FITC, CD54‐Percp‐cy_5.5_, CD54‐PE, and CD45‐BV_510_ (all antibodies were purchased from BD Biosciences, San Diego, CA, USA). DAPI was used to identify and sort the dead cells. The remaining steps were the same as the protocol described in our previous report 19. The absolute CTCs or antibody‐positive cell numbers were derived from the absolute number of white blood cells provided by the hematological analyzer, and the percentage of CTCs or antibody‐positive cells was determined by flow cytometry, using the following formula: percentage of cells × white blood cells count/100.

### Clinical and survival information

A total of 152 CRC patients who underwent surgery or treatment from December 2013 to October 2014 in our department were prospectively evaluated. All CRC patients with liver metastasis received the appropriate treatment, surgical treatment or chemotherapy, which involved discussion with a multidisciplinary team. The surgical treatment for liver metastasis included resection, radiofrequency ablation, and transcatheter arterial chemoembolization. All patients were scheduled for periodic follow‐ups. Disease recurrence is defined as local (colon, pelvis, or peritoneum) or systemic (hepatic, pulmonary, other organ, or multiorgan) on the basis of clinical, endoscopic, or radiological findings.

### Statistical analyses

Continuous variables were expressed as the mean ± standard deviation, and the explorative comparison of independent groups was performed by the *t‐*test for normal distributions, and the Mann–Whitney *U*‐test (two groups) or the Kruskal–Wallis test (more than two groups) for nonparametric distributions. The distribution of nominal‐ or ordinal‐scaled variables was compared using Pearson's *χ* ^2^ test. Time‐dependent survival probabilities were estimated using the Kaplan–Meier method, and the log‐rank (Mantel–Cox) test was used to compare independent subgroups of CRC patients with or without liver metastasis. Disease‐free survival (DFS) and overall survival (OS) were used as the primary outcome parameters. DFS was calculated from the date of surgery until the date when a recurrence or metastasis first occurred for CRC patients without distant metastasis. All statistical tests were two‐sided, and the value of *P *<* *0.05 was considered to be statistically significant. All statistical analyses were performed using SPSS Statistics software for Windows, version 22 (SPSS, Chicago, IL, USA).

## Results

### Clinicopathological features and definition of the cellular subpopulation of CTCs

Between December 2013 and October 2014, 152 CRC patients who underwent surgical treatment or received chemotherapy in our department were prospectively evaluated. The majority of CRCs (nearly 50%) occurred in patients aged 40–64 years, and the proportion of male patients was nearly 20% higher than that of female patients. Among the 78 CRC patients with liver metastasis, 24 patients underwent resection for primary tumor together with surgical treatment for metastatic tumor after discussions with the multidisciplinary team; another 32 patients underwent only resection for primary tumor. In addition to surgical treatment, these 56 patients also received chemotherapy; the remaining 22 patients received chemotherapy only (Table 1).

**Figure 1:**
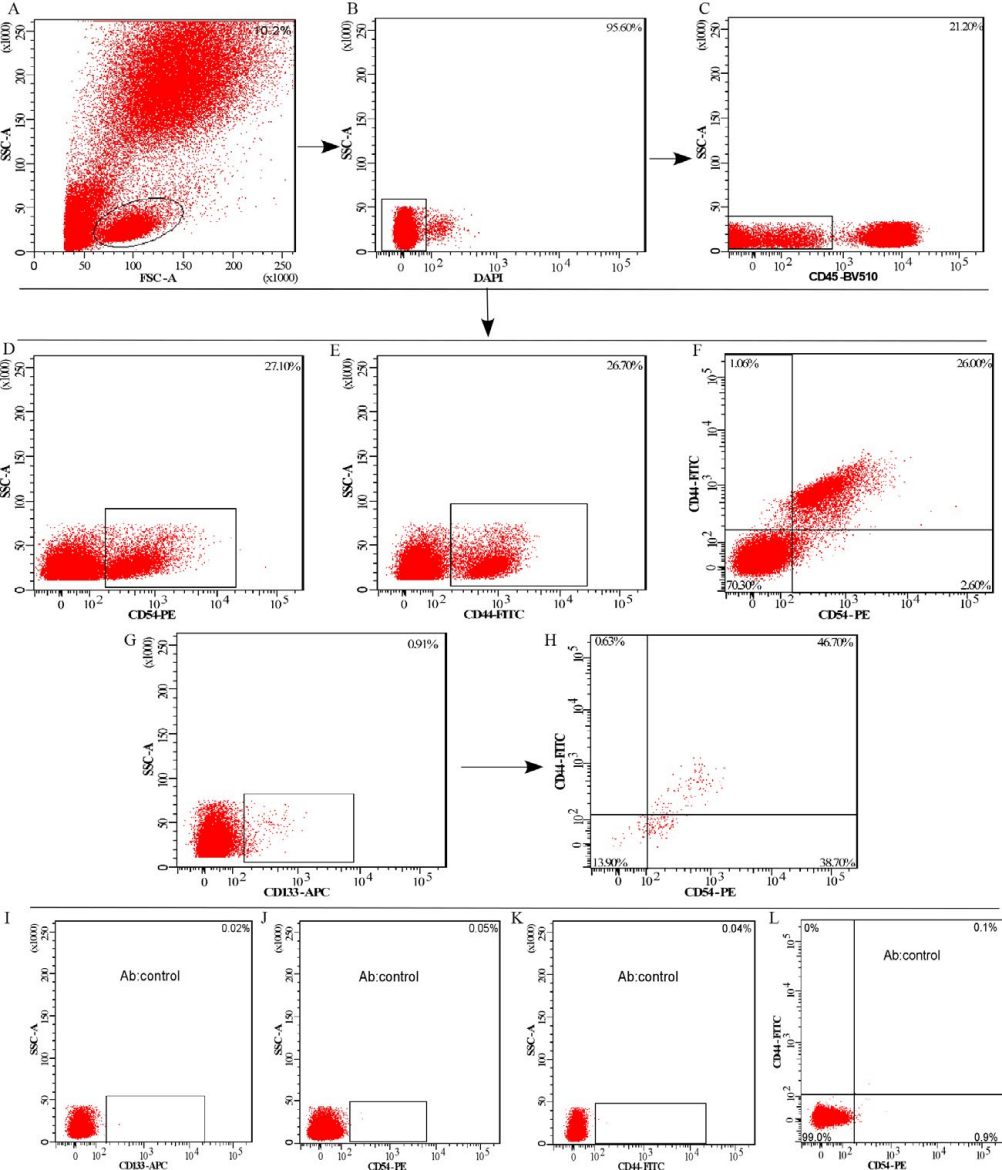
Expression of the cellular subpopulation of circulating tumor cells in the peripheral blood of circulating tumor cells (CTCs). (A) The gating strategy to detect cellular subpopulations in whole blood or blood depleted of hematopoietic cells by fluorescence‐activated cell sorting (FACS). (B, C) The gating strategy to sort DAPI ^−^ CD45^−^ cells, (D–H) the plots are gated on DAPI ^−^ CD45^−^ cells. The contour plots show the expression of CD54^+^, CD44^+^, CD54^+^ CD44^+^, CD133^+^, and CD133^+^ CD54^+^ CD44^+^ cellular subpopulations of CTCs in the peripheral blood of colorectal cancers, respectively. The percentages of cells are indicated for each gate or plot quadrant. (I–L) The AB control for each marker, respectively, or individually.

The expression of the CTC surface markers, including CD133, CD54, and CD44, in the peripheral blood were measured using FACS. The protocol to sort the cellular subpopulations of CTCs has been reported in our previous study, and is described in Figure 1. A total of 50 cases were then randomly chosen as the training group, and the median of expression for each single marker or the combination of markers was set as the cutoff point to divide the patients into two groups (high and low expression).

**Table 1:** Clinicopathological characteristics of the patients

	*N* = 152	100%
Sex		
Men	90	59.2%
Women	62	40.8%
Age, years (median)	61.59 ± 12.08	
Tumor location		
Rectum	106	69.7%
Left hemicolon	17	11.2%
Right hemicolon	29	19.1%
Serum CEA level^a^ (*n*)		
0	99	65.1%
1	37	24.3%
2	16	10.5%
Serum CA19‐9 level^b^ (*n*)		
0	121	79.6%
1	16	10.5%
2	15	9.9%
Stage		
I	15	9.9%
II	31	20.4%
III	28	18.4%
IV	78	51.3%
Treatment of metastatic CRC (*N* = 78)		
No surgical treatment	22	28.2%
Surgical treatment for primary tumor	32	41.0%
Surgical treatment for primary and metastatic tumor	24	30.8%
Recurrence^c^(local and distant)		
No	61	62.2%
Yes	37	37.8%
Survival status		
Alive	92	60.5%
Death	60	39.5%

a CEA: 0 < 5 ng/mL, >5 to <20 ng/mL, 2 > 20 ng/mL.

b CA19‐9: 0 < 20 ng/mL, 1 > 20 to <50 ng/mL, 2 > 50 ng/mL.

c Including metastatic patients who received the surgical treatment for primary and metastatic tumor.

### Survival distribution and the analysis for CRCs without distant metastasis

The median survival during the follow‐up was 36 months (range, 31–43 months). No patients died during the first month after the surgery because of postoperative complications or were lost during the study years. Thirty‐seven patients developed a local recurrence or distant metastasis, including 16 metastatic patients who underwent resection for primary tumor together with surgical treatment for metastasis. Sixty patients died during the follow‐up. We then performed the survival analyses for the subgroup of CRC patients with liver metastasis and CRC patients without distant metastasis. First, concerning the DFS and OS, patients with a high level of CEA or with a positive risk factor had a worse survival than those without CRCs without distant metastases. However, only a few cellular subpopulations of CTCs had a significant effect on survival, and multivariable analyses showed that there was no cellular subpopulation of CTCs that had a prognostic value among these patients (Table S1).

### Surgical treatment of metastases, the CEA levels, and CD133^+^CD44^+^CD54^+^ subpopulation of CTCs had a significant effect on the survival of CRCs with liver metastasis

**Figure 2:**
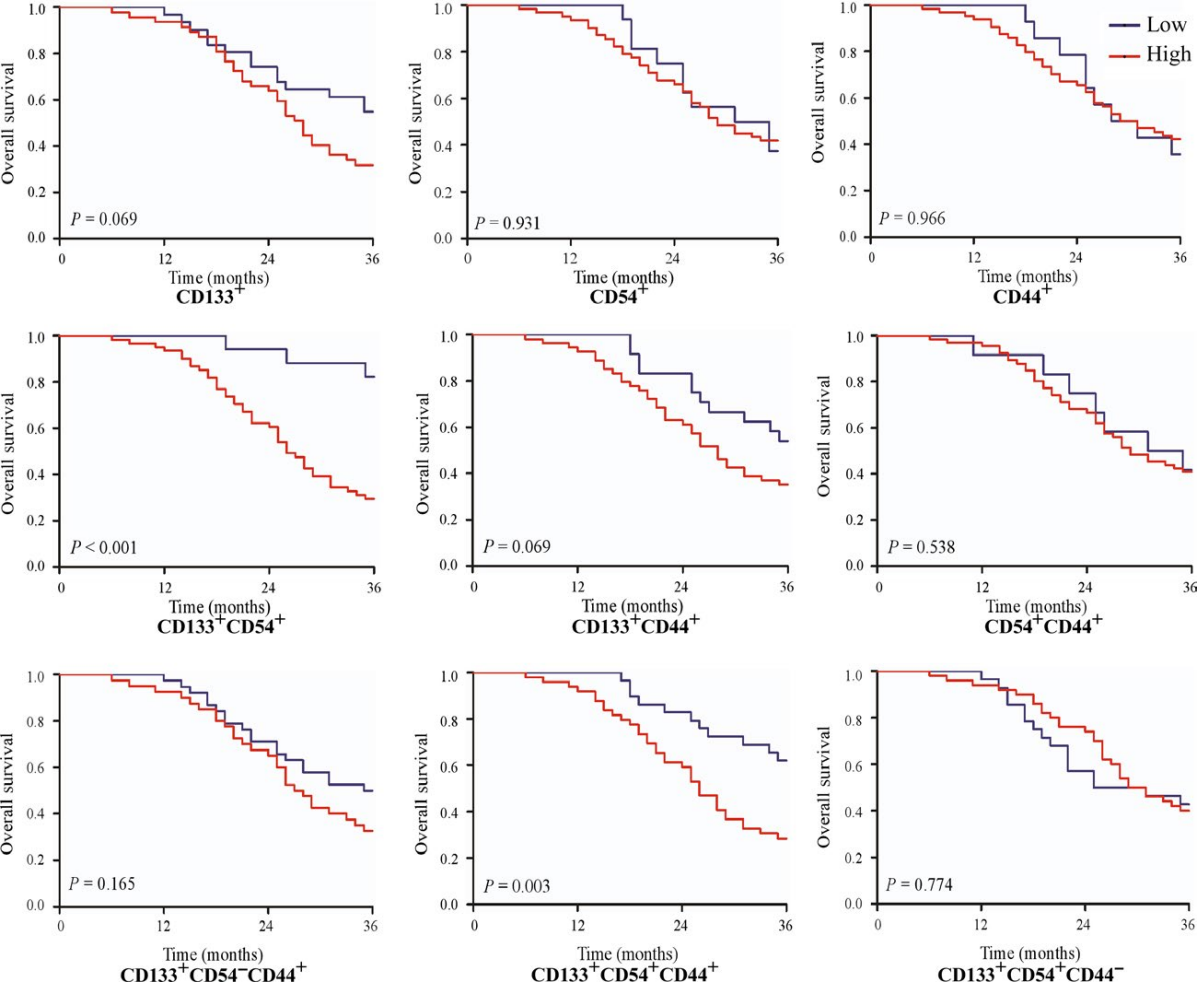
The overall survival of CRCs with liver metastasis with high and low expressions of different cellular subpopulations of circulating tumor cells with the cellular markers CD133, CD54, and CD44, respectively. The significance of differences between the survival curves was calculated by the log‐rank test. The abbreviations are the same as in Figure 1 legend. CRCs = colorectal cancer cells.

**Figure 3:**
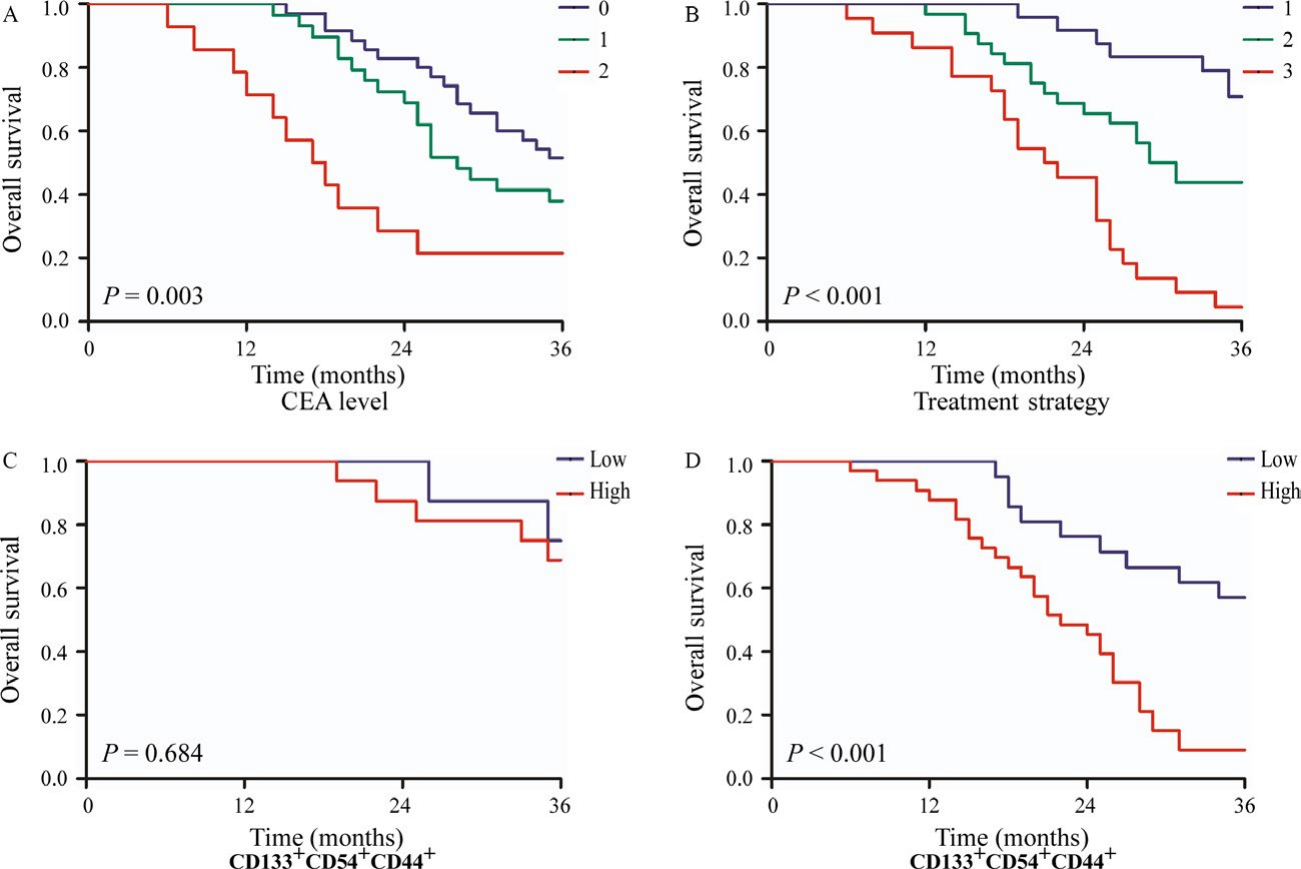
The overall survival of CRCs with liver metastasis in different subgroups. (A) Survival curves of CRCs with liver metastasis according to the subgroup of serum carcinoembryonic antigen levels. 0, <5 ng/mL; 1, 5–20 ng/mL; 2, >20 ng/mL. (B) Survival curves of CRCs with liver metastasis according to the subgroup of treatment strategy. (1) Patients who underwent resection for primary tumor accompanied by surgical treatment for metastasis; (2) patients who only underwent resection for primary tumor; and (3) patients who received chemotherapy only. The significance of differences between survival curves was calculated by the log‐rank test. (C) Survival curves of CRC patients with liver metastasis who underwent surgical treatment for liver metastasis. (D) Survival curves of CRC patients with liver metastasis who did not undergo surgical treatment for liver metastasis. The significance of differences between survival curves was calculated by the log‐rank test. The abbreviations are the same as in Figures 1 and 2 legends.

Among the CRC patients with liver metastasis, surgical treatment for metastasis had a significant effect on the survival, because patients who underwent resection for primary tumor together with surgical treatment for metastasis had a better survival than those who did not undergo surgical treatment for the metastases (3‐year OS, 70.8%, 43.8%, and 4.5%, respectively; *P *<* *0.001; Table 2; Fig. 3). Patients with a higher level of CEAs had a significantly worse survival than those who had lower CEA levels (3 year OS, 21.4%, 37.9%, and 51.4% for high, middle, and low level, respectively; *P *=* *0.003; Table 2; Fig. 3). A similar result was found among patients with lymphovascular invasion (3 year OS, 15.7% vs. 61.3%; *P *=* *0.009; Table 2). However, sex, location of the primary tumor, or the number of metastases also had a significant effect on survival. We then analyzed the prognostic value of cellular subpopulations of CTCs. Concerning the OS, patients with a high expression of CD133^+^CD54^+^ (*P *<* *0.001), CD133^−^CD54^+^ (*P *=* *0.004), and CD133^+^CD44^+^CD54^+^ (*P *=* *0.003) of CTCs had a worse survival than those with a low expression. Table 2 and Figure 2 show additional results of the survival analyses.

**Table 2:** Results of stepwise Cox multivariate regression models for OS of CRC patients with liver metastasis

Covariate	Univariate	Multivariate	
	*P*‐value	*P*‐value	HR (CI)
Gender	0.017		
Age (65 years)	0.083		
Tumor location	0.149		
Numbers of liver metastases	0.322		
CEA level	0.003	0.007	3.056 (1.354–6.897)
CA19‐9 level	0.469		
Extra‐nodal tumor deposits	0.049		
Lymphovascular invasion	0.009	0.054	3.055 (0.981–9.512)
Ascites	0.165		
Obstruction	0.766		
Treatment strategy	<0.001	0.023	0.212 (0.056–0.808)
CD133^+^ subpopulation	0.069		
CD54^+^ subpopulation	0.931		
CD44^+^ subpopulation	0.966		
CD133^+^CD44^−^ subpopulation	0.962		
CD133^+^CD44^+^ subpopulation	0.069		
CD133^−^CD44^+^ subpopulation	0.206		
CD133^+^CD54^−^ subpopulation	0.981		
CD133^+^CD54^+^ subpopulation	<0.001	0.079	4.838 (0.834–28.078)
CD133^−^CD54^+^ subpopulation	0.004		
CD54^+^CD44^−^ subpopulation	0.437		
CD54^+^CD44^+^ subpopulation	0.538		
CD54^−^CD44^+^ subpopulation	0.542		
CD133^+^CD44^+^CD54^−^ subpopulation	0.165		
CD133^+^CD44^+^CD54^+^ subpopulation	0.003	0.014	6.459 (1.461–28.558)
CD133^+^CD44^−^CD54^+^ subpopulation	0.774		

Multivariable survival analyses of CRC patients with liver metastasis are shown in Table 2. When potential variables (*P *<* *0.1) were considered, CEA levels (hazard ratio [HR] = 3.056; 95% confidence interval [CI] = 1.354–6.897; *P *=* *0.007), treatment strategy (HR = 0.212; 95% CI = 0.056–0.808; *P *=* *0.023), and CD133^+^CD44^+^CD54^+^ subpopulations of CTCs (HR = 6.459; 95% CI = 1.461–28.558; *P *=* *0.014) were independent prognostic factors for CRC patients with liver metastasis. Furthermore, survival analyses were performed for two subgroups of patients who underwent resection for primary tumors accompanied by surgical treatment for metastasis, and patients who did not undergo surgical treatment for metastases. Patients who did not undergo surgical treatment for metastases with a high expression of CD133^+^CD44^+^CD54^+^ subpopulation of CTCs had a worse survival than those with a low expression (3 year OS, 9.1% vs. 57.1%; *P *<* *0.001; Fig. 3). However, the expression of CD133^+^CD44^+^CD54^+^ subpopulation of CTCs did not affect the survival of patients who had resection for primary tumor accompanied by surgical treatment for metastasis (*P *=* *0.684; Fig. 3).

## Discussion

Liver metastasis is one of the most important prognostic factors for CRC, and increasing evidence indicates that synchronous metastatic colorectal liver disease is associated with a disseminated disease state and a worse prognosis 5, 20. In the recent decades, the advances in the treatment such as new therapies including antiepidermal growth factor receptor antibody therapy and antiangiogenic agents have only partially improved the survival of CRC patients with distant‐stage disease, with a 2‐year relative survival rate increased from 21% to 35% for colon cancer with distant stage and from 22% to 39% for rectal cancer with distant stage between 1989–1992 and 2009–2012 5, 20, 21, 22. The identification of prognostic factors is the critical for improving the survival of CRC patients with synchronous liver metastasis.

This study showed that surgical treatment for liver metastasis was an independent prognostic factor for CRC patients with liver metastasis (HR = 0.212; 95% CI = 0.056–0.808; *P *=* *0.023) as patients who underwent resection for primary tumor together with surgical treatment for metastasis had a better survival than other hepatic metastatic patients (3 year OS, 70.8%, 43.8%, and 4.5%; *P *<* *0.001; Table 2; Figure 3). This result is consistent with the European consensus, which emphasizes the importance of achieving R0‐resection, either initially or after induction treatment for both metastatic disease and primary tumor after a multidisciplinary team discussion 6, 9, 23. Another study reported that local surgical therapies for metastases, including hepatic arterial infusion, radioembolization, and transcatheter arterial chemoembolization affected the survival of the metastatic CRC patients 7, 24. We also found that the level of CEAs had a significant effect on the survival of CRC patients with liver metastasis (HR = 3.056; 95% CI = 1.354–6.897; *P *=* *0.007). The level of CEAs reflects the degree of cancer disease and provides a reference for the diagnosis of the distant‐stage disease, evaluation of the prognosis, and monitoring the recurrence during the follow‐up 25, 26.

In a further analysis of the prognostic value of the cellular subpopulation of CTCs, we found that CRC patients with high expression of CD133^+^CD54^+^ (*P *<* *0.001), CD133^−^CD54^+^ (*P *=* *0.004), and CD133^+^CD44^+^CD54^+^ (*P *=* *0.003) subpopulations of CTCs had a worse survival than those with low expression. It was further showed that CD133^+^CD44^+^CD54^+^ subpopulation of CTCs was an independent prognostic factor for CRC patients with liver metastasis (HR = 6.459; 95% CI = 1.461–28.558; *P *=* *0.014). In our previous research, we had reported that CD133^+^CD44^+^CD54^+^ subpopulation of CTCs in the peripheral blood was associated with liver metastasis and could be used as an auxiliary diagnostic marker for liver metastasis among CRC patients 19. This is the first study that reports CD133^+^CD44^+^CD54^+^ subpopulation of CTCs has a prognostic value in CRC patients with liver metastases, especially those who did not receive surgical treatment for metastases (*P *<* *0.001).

Cancer initiating cells (CICs) are referred as a rare cellular subpopulation of CTCs with properties of self‐renewal, tumor‐initiating, motile and invasive, increased resistance to apoptosis and are important in facilitating metastasis 10. Previously, cellular subpopulations from tumor tissue such as CD54^+^CD44^+^ 18, CD26^+^ 27, CD133^+^CD44^+^ 28, and CD133^+^CXCR4^+^ 29 had been sorted and identified as CICs or MICs. The existence and phenotype of MICs was first reported in the peripheral blood of primary human luminal breast cancer using a xenograft assay 30. CD133 has been accepted as a cancer initiating cell marker for colon cancer and it has been shown that CD133^+^ cellular subpopulation could be used as a baseline to sort and detect CTCs in the peripheral blood of CRC patients using FACS. The expression of CD133^+^CD54^+^CD44^+^ cellular subpopulation of CTCs was significantly associated with liver metastasis and was an independent prognostic factor for CRCs with liver metastasis 19. Whether CD133^+^CD54^+^CD44^+^ cellular subpopulation of CTCs involved CICs or MICs in the peripheral blood still needs further study.

In conclusion, we showed that CD133^+^CD44^+^CD54^+^ subpopulation of CTCs has a prognostic value in CRC with liver metastasis, and has a significant effect on the survival of CRC patients who did not undergo surgical treatment for metastasis. Based on these observations, further study needs to be carried out to investigate the molecular characterization and metastatic capacity of CD133^+^CD44^+^CD54^+^ cellular subpopulation of peripheral blood.

## Conflict of Interest

None declared.

## Supporting information


**Table S1.** Results of stepwise Cox multivariate regression models for DFS and OS of CRC patients without metastasis.
