# Supplementary material for: Prognostic value of CD133+ CD54+ CD44+ circulating tumor cells in colorectal cancer with liver metastasis
Source: Cancer Med. 2017 Nov 3;6(12):2850–7. doi: 10.1002/cam4.1241 (PMC5727299; doi:10.1002/cam4.1241)
Supplement: Supplementary file 1 — Table S1. Results of stepwise Cox multivariate regression models for DFS and OS of CRC patients without metastasis. [file CAM4-6-2850-s001.docx]

Supplementary Table 1. Results of Stepwise Cox Multivariate Regression Models for DFS and OS of CRC patients without metastasis

| Covariate | DFS | | | OS | | |
| --- | --- | --- | --- | --- | --- | --- |
|  | Univariate | Multivariate | | Univariate | Multivariate | |
|  | P-value | P-value | HR(CI) | P-value | P-value | HR(CI) |
| Gender | 0.012 |  |  | 0.807 |  |  |
| Age(65 years) | 0.061 |  |  | 0.574 |  |  |
| Tumor location | 0.115 |  |  | 0.710 |  |  |
| Stage | 0.048 |  |  | 0.092 |  |  |
| CEA level | <0.001 |  |  | <0.001 | 0.003 | 8.175(2.007-33.306) |
| CA19-9 level | 0.040 |  |  | 0.357 |  |  |
| Extra nodal tumor deposits | 0.049 |  |  | 0.006 |  |  |
| lymphovascular invasion | 0.158 | 0.067 | 4.434(0.901-21.828) | 0.677 |  |  |
| Ascites | 0.551 |  |  | 0.205 |  |  |
| Obstruction | 0.305 |  |  | 0.293 |  |  |
| CD133^+^ subpopulation | 0.254 |  |  | 0.251 |  |  |
| CD54^+^ subpopulation | 0.037 |  |  | 0.193 |  |  |
| CD44^+^ subpopulation | 0.067 |  |  | 0.795 |  |  |
| CD133^+^CD44^-^ subpopulation | 0.140 |  |  | 0.884 |  |  |
| CD133^+^CD44^+^ subpopulation | 0.142 |  |  | 0.950 |  |  |
| CD133^-^CD44^+^ subpopulation | 0.146 |  |  | 0.169 |  |  |
| CD133^+^CD54^-^ subpopulation | 0.220 |  |  | 0.771 |  |  |
| CD133^+^CD54^+^ subpopulation | 0.037 |  |  | 0.193 |  |  |
| CD133^-^CD54^+^ subpopulation | 0.009 |  |  | 0.274 |  |  |
| CD54^+^CD44^-^ subpopulation | 0.242 |  |  | 0.481 |  |  |
| CD54^+^CD44^+^ subpopulation | 0.098 |  |  | 0.975 |  |  |
| CD54^-^CD44^+^ subpopulation | 0.227 |  |  | 0.152 |  |  |
| CD133^+^CD44^+^CD54^-^ subpopulation | 0.954 |  |  | 0.489 |  |  |
| CD133^+^CD44^+^CD54^+^ subpopulation | 0.113 |  |  | 0.872 |  |  |
| CD133^+^CD44^-^CD54^+^ subpopulation | 0.078 |  |  | 0.241 |  |  |
